# Influenza mRNA vaccine reduces pathogenicity and transmission of A(H5N1) virus in a ferret model

**DOI:** 10.1038/s41541-025-01318-3

**Published:** 2025-11-29

**Authors:** Masato Hatta, Nicole Brock, Teresa Hauguel, Chenchen Feng, Ying Huang, Jana M. Ritter, Yasuko Hatta, Matthew W. Keller, Ivna De Souza, Jaber Hossain, Elizabeth A. Pusch, Thomas Rowe, Herg Zhang, Liyang Cui, Sarah O’Leary, Juan A. De La Cruz, Monique C. Johnson, Jessica A. Belser, Xiangjie Sun, Jimma Liddell, Margaret Creech, Joseph R. Rouse, Paul Carney, Jessie Chang, Michael Currier, Li Wang, Marie K. Kirby, Han Di, John R. Barnes, James Stevens, Vivien G. Dugan, C. Todd Davis, David E. Wentworth, Pirada Suphaphiphat Allen, Taronna R. Maines, Bin Zhou

**Affiliations:** 1 Influenza Division, National Center for Immunization and Respiratory Diseases, Centers for Disease Control and Prevention, Atlanta, GA USA; 2 Pfizer, Inc, Pearl River, NY USA; 3 Division of High-Consequence Pathogens and Pathology, National Center for Emerging and Zoonotic Infectious Diseases, Centers for Disease Control and Prevention, Atlanta, GA USA; 4 Oak Ridge Institute of Science and Education (ORISE), Oak Ridge, TN USA; 5 Office of Advanced Molecular Detection, National Center for Emerging and Zoonotic Infectious Diseases, Centers for Disease Control and Prevention, Atlanta, GA USA; 6 Present Address: GSK, Cambridge, MA, USA

**Keywords:** Diseases, Immunology, Microbiology

## Abstract

The global spread of highly pathogenic avian influenza A(H5N1) viruses poses a serious pandemic threat. While sustained human-to-human transmission has not occurred, widespread circulation in birds, increased detection in mammals, and occasional human spillovers underscore the need for safe and effective vaccines. We evaluated an H5 mRNA vaccine candidate in ferrets using recent clade 2.3.4.4b A(H5N1) human isolates. Vaccination elicited strong neutralizing antibodies, conferred robust protection against lethal challenge, and significantly reduced viral titers. In a direct contact transmission model, mRNA vaccination decreased virus shedding in inoculated ferrets and reduced onward transmission; it also protected vaccinated contact ferrets from infection following exposure to virus-shedding, unvaccinated ferrets. Additionally, sera from vaccinated animals cross-neutralized clade 2.3.2.1e human viruses to varying degrees, depending on the strain. These findings demonstrate that H5 mRNA vaccination not only protects against disease but also reduces transmission, supporting its potential as a key tool for pandemic preparedness.

## Introduction

Highly pathogenic avian influenza (HPAI) A(H5) viruses pose a serious pandemic threat due to their worldwide spread among wild birds and poultry, and sporadic cases among humans. Since November 2003, HPAI A(H5N1) has been confirmed in 969 human cases across 24 countries, 467 of which were fatal^1^. Starting in 2021, clade 2.3.4.4b HPAI A(H5N1) viruses spread extensively among wild and domestic birds to broad geographic regions, including Asia, Africa, Europe, the Middle East, the Americas, and Antarctica^2–6^. As a result, an unprecedented number of deaths among wild birds and poultry have been reported^3,7–10^. The clade 2.3.4.4b HPAI A(H5N1) virus has also been detected in a wide range of mammalian species^11,12^.

In 2022–2023, Chile reported outbreaks of clade 2.3.4.4b HPAI A(H5N1) virus among backyard poultry and farmed poultry, wild birds, and sea mammals^13–15^ and reported its first confirmed human infection of A(H5N1) on March 29, 2023^16,17^. On March 25, 2024, the U.S. state of Texas reported an outbreak of clade 2.3.4.4b HPAI A(H5N1) virus in dairy cattle. This is the first time that HPAI viruses had been found in cows. This clade of virus subsequently spread to 17 states, with 1,031 infected dairy herds reported as of April 21, 2025^18^. The first human case linked to dairy cattle exposure was reported on April 1, 2024, in Texas^19,20^. As of March 24, 2025, 70 human cases and one fatality have been reported in the U.S., most of which were exposed to infected or potentially infected dairy herds or poultry^21,22^. Although human infections with clade 2.3.4.4b HPAI A(H5N1) viruses remain relatively rare and no human-to-human transmission has been reported in the U.S., the unprecedented and sustained spread in mammals increases the risk of adaptive mutations that could enable human-to-human transmission and potentially trigger a pandemic.

Vaccination remains the most effective strategy to control and prevent influenza virus infection in people. Three A(H5N1) vaccines are approved by the U.S. Food and Drug Administration (FDA), which use inactivated A(H5N1) virus strains from 2004–2005^23^, although none are currently commercially available or recommended for public use. These vaccines differ from the circulating clade 2.3.4.4b viruses by approximately 30–45 amino acids in the hemagglutinin (HA) protein and may not offer sufficient protection. While inactivated vaccines targeting clade 2.3.4.4b viruses are under development, the advantages of the mRNA vaccine platform, such as rapid development and scalable production, offer a valuable complement to traditional influenza vaccine strategies.

In this study, we evaluated an A(H5) mRNA vaccine developed using the Pfizer platform. Using a ferret model, we assessed its ability to protect against disease and reduce transmission following challenge with two clade 2.3.4.4b HPAI A(H5N1) viruses isolated from human cases in Chile and the U.S.

## Results

### Immunogenicity of an H5 mRNA vaccine candidate in ferrets

A nucleoside-modified mRNA construct encoding the full-length, codon-optimized HA protein with the polybasic cleavage site deleted from A/Astrakhan/3212/2020 A(H5N8) (Ast/20), a WHO-recommended prototype candidate vaccine virus (CVV), was synthesized and formulated in lipid nanoparticles (LNPs) (Supplementary Fig. 1A). Production and quality control followed the same platform methods used for Pfizer’s COVID-19 and seasonal influenza mRNA vaccines^24–26^. In vitro analysis confirmed that HA protein expression was efficient and dose-dependent (Supplementary Fig. 1B).

**Fig. 1:**
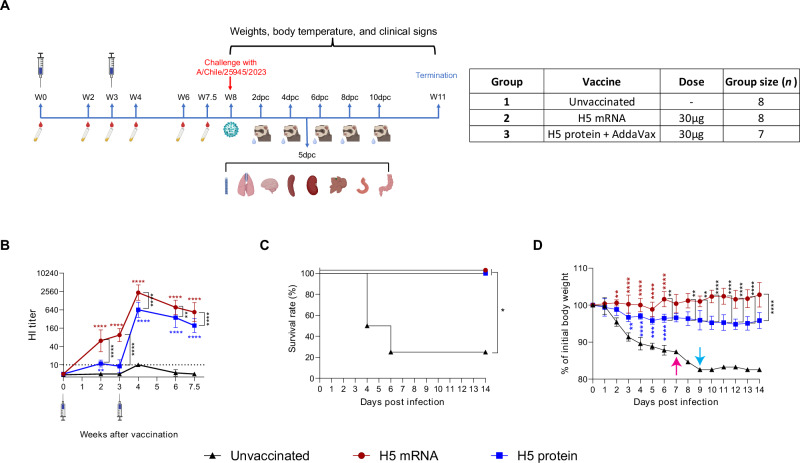
H5 mRNA vaccine candidate elicited strong antibody responses and protected ferrets from lethal challenge with an HPAI A(H5N1) virus. **A** Ferrets (*n* = 7–8 per group) were vaccinated intramuscularly twice, three weeks apart, with 30 μg of mRNA vaccine encoding HA from Ast/20 (Group 2) or 30 μg of recombinant Ast/20 H5 HA protein adjuvanted with AddaVax (Group 3). Control animals received PBS only (Group 1). Five weeks after the second vaccination, ferrets were challenged with clade 2.3.4.4b HPAI A(H5N1) Chile/23 virus. Nasal wash samples were collected on days 2, 4, 6, 8, and 10 post-challenge. Four animals from Group 1 and 2, and three animals from Group 3 were euthanized on day 5 post-challenge and organs were collected for virus quantification and histopathology analysis. **B** Serum samples were collected from ferrets 2, 3, 4, 6 and 7.5 weeks following the first vaccination and HI titers were measured using Ast/20 CVV with 1% HRBC. Titers are reported as geometric mean titer with geometric SD. The dashed line indicates the limit of detection for the assay (10 HI). **C** Survival was monitored for 14 days post-challenge. The survival rate was calculated with 4 ferrets which were not used for organ sampling. **D** The body weight of ferrets was monitored daily for 14 days post-challenge. The body weight changes were calculated with 4 ferrets which were not used for organ sampling. Since three ferrets in the unvaccinated group were euthanized due to severe disease symptoms by day 6, the body weight data of the one remaining ferret is shown thereafter (pink arrow). As that ferret continued to lose weight, a carnivore plus diet was given on day 9 (cyan arrow). Asterisk color coding in (**B**) and (**D**): red = H5 mRNA vs. unvaccinated; blue = H5 protein vs. unvaccinated; black = H5 mRNA vs. H5 protein. *P < 0.05; **P < 0.01; ***P < 0.001; ****P < 0.0001.

To evaluate antibody responses, ferrets (*n* = 7 or 8 per group) were vaccinated intramuscularly twice, three weeks apart, with PBS (Group 1), 30 μg H5 mRNA vaccine (Group 2), or 30 μg recombinant Ast/20 HA protein with AddaVax adjuvant (Group 3) (Fig. 1A). Serum was collected at baseline and at 2, 3, 4, 6, and 7.5 weeks after the first dose. Antibody titers were measured by hemagglutination inhibition (HI) assay using 1% horse red blood cells (HRBC), which are more sensitive than turkey red blood cells for detecting HI antibodies against avian influenza viruses, including clade 2.3.4.4b strains^27,28^. A single dose of the H5 mRNA vaccine significantly increased HI titers (*P* < 0.0001), whereas the adjuvanted protein vaccine did not (Fig. 1B). After two doses, mean HI titers reached 2345 in mRNA-vaccinated ferrets and 640 in protein-vaccinated animals, with the mRNA group showing significantly higher titers at all post-vaccination time points (*P* < 0.0001 or *P* < 0.01).

### H5 mRNA vaccine candidate protects ferrets against lethal challenge with a clade 2.3.4.4b HPAI A(H5N1) virus

To evaluate the efficacy of the H5 mRNA vaccine candidate, ferrets were challenged intranasally with 10^4^ TCID_50_ of HPAI A(H5N1) A/Chile/25945/2023 (Chile/23) virus, five weeks after the second vaccination (8 weeks after the first dose). All ferrets vaccinated with H5 mRNA (Group 2) or adjuvanted H5 protein (Group 3) survived (4/4), while only one of four unvaccinated ferrets (Group 1) survived (Fig. 1C). The surviving unvaccinated ferret lost nearly 20% of its body weight but remained above the humane endpoint threshold (Fig. 1D). Despite receiving supportive care, including carnivore plus diet feeding on day 9 post-challenge, the animal failed to regain weight (supportive care was not provided to any other vaccinated or unvaccinated ferrets). In contrast, H5 mRNA-vaccinated ferrets exhibited minimal weight loss (mean maximum: 1.2%), while protein-vaccinated ferrets lost slightly more (mean maximum: 5.3%). The difference in weight loss between the mRNA and protein vaccine groups was statistically significant (*P* < 0.05) (Fig. 1D). These results indicate that the H5 mRNA vaccine candidate effectively protected ferrets against HPAI A(H5N1) disease, reducing severity and preventing lethal outcomes.

### H5 mRNA vaccine candidate reduces viral replication in the respiratory tract and intestines

**Fig. 2:**
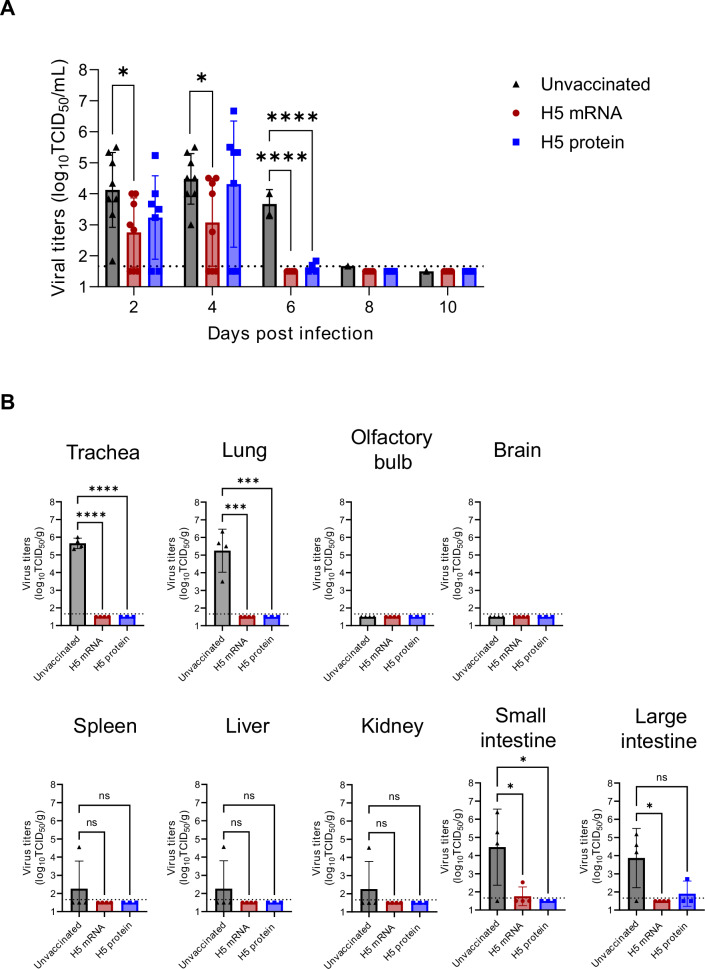
H5 mRNA vaccine candidate reduced HPAI A(H5N1) virus replication in nasal wash and organs of challenged ferrets. **A** The Chile/23 viral titers in nasal wash samples were determined by TCID_50_ assay. *n* = 8 (Group 1 and 2) and *n* = 7 (Group 3) on day 2 and 4 post-challenge and *n* = 4 thereafter as 4 ferrets (Group 1 and 2) and 3 ferrets (Group 3) were euthanized for organ sampling on day 5 post-challenge. Data are presented as geometric means with geometric standard deviations (SD). Dashed lines indicate the limit of detection (1.67 log_10_TCID_50_/mL) for the assays. **B** Organ samples were collected from 4 ferrets (Group 1 and 2) or 3 ferrets (Group 3) on day 5 post-challenge and homogenized for virus titration by TCID_50_ assay. Data are presented as geometric means with geometric standard deviations (SD). Dashed lines indicate the limit of detection (approximately 1.67 log_10_TCID_50_/g) for the assays. Samples below the LOD were assigned a value of 1.5 for mean calculation and statistical testing. *P < 0.05; ***P < 0.001; ****P < 0.0001; ns = not significant.

To determine the effect of H5 mRNA vaccine on viral replication, we determined viral titers in nasal wash (NW) samples collected at 2, 4, 6, 8, and 10 days post-challenge (dpc) using a TCID_50_ assay (2–4 dpc, *n* = 7–8; thereafter *n* = 4 because select animals were euthanized at 5 dpc for organ sampling) (Fig. 2A). H5 mRNA-vaccinated ferrets (Group 2) exhibited significantly lower viral titers than unvaccinated ferrets (Group 1) from 2 through 6 dpc (*P* < 0.05), with complete viral clearance by 6 dpc. Protein-vaccinated ferrets (Group 3) cleared virus by day 8, but viral titers did not differ significantly from controls (Fig. 2A).

To evaluate viral dissemination, tissues were collected on day 5 from select animals and infectious virus was quantified (Fig. 2B). In unvaccinated ferrets, robust virus replication was detected in the trachea and lungs of all four ferrets; in the spleen and kidney of one ferret; in the liver of one ferret; and in the small and large intestines of three ferrets. In contrast, virus was detected only in the small intestine of one mRNA-vaccinated ferret and in the large intestine of one protein-vaccinated ferret. No virus was detected in the trachea, lung, spleen, liver, or kidney of vaccinated ferrets. Viral titers in the olfactory bulb and brain were below detection in all groups (Fig. 2B). Digital PCR confirmed significantly lower RNA levels in the trachea and lungs of vaccinated ferrets (*P* < 0.001), consistent with infectious titers (Supplementary Fig. 2). These results demonstrate that the H5 mRNA vaccine markedly reduced viral replication in both the upper and lower respiratory tracts and intestines.

### H5 mRNA vaccine candidate mitigates lung pathology and prevents pneumonia

Histopathologic analysis of respiratory tissues revealed marked protection from lung pathology in H5 mRNA-vaccinated ferrets compared to unvaccinated controls (Supplementary Fig. 3). Among unvaccinated animals, two had lung lobes with up to 50% of the parenchyma affected by pneumonic changes. Affected areas had peribronchial and peribronchiolar lymphoplasmacytic inflammation, and variable epithelial necrosis and luminal filling by degenerate inflammatory cells, neutrophils, and/or mucus. Alveoli were filled and sometimes effaced by neutrophils, macrophages, and few erythrocytes. Non-pneumonic lung regions showed mild congestion and mild interstitial and perivascular mononuclear infiltrates. The other two unvaccinated animals showed milder but similar changes affecting 10–20% of the examined lung lobes. In all unvaccinated ferrets, A(H5N1) viral antigen was detected by immunohistochemistry (IHC) in bronchial and bronchiolar epithelium, including bronchial submucosal glands, and in alveolar epithelium and inflammatory cells within the pneumonic lung tissue.

Among the four H5 mRNA-vaccinated animals, two had patchy foci of peribronchial, and more prominent peribronchiolar, and dense perivascular inflammation. Inflammation was comprised mostly of lymphocytes, plasma cells, and histiocytes, but neutrophils were occasionally present within and around bronchiolar lumens. Alveolar walls contained low numbers of mononuclear cells, but alveolar lumina were free of inflammation. Lung from a third H5 mRNA-vaccinated animal had only bronchial submucosal inflammation, and the lung from the fourth animal was unremarkable. No viral antigen was detected by IHC in conducting airways or lung parenchyma of H5 mRNA-vaccinated animals. Ferrets vaccinated with the adjuvanted H5 protein displayed similar or slightly greater inflammation than mRNA-vaccinated animals, without viral antigen detection.

### H5 mRNA vaccine candidate reduces transmission of a clade 2.3.4.4b HPAI A(H5N1) virus in ferrets

**Fig. 3:**
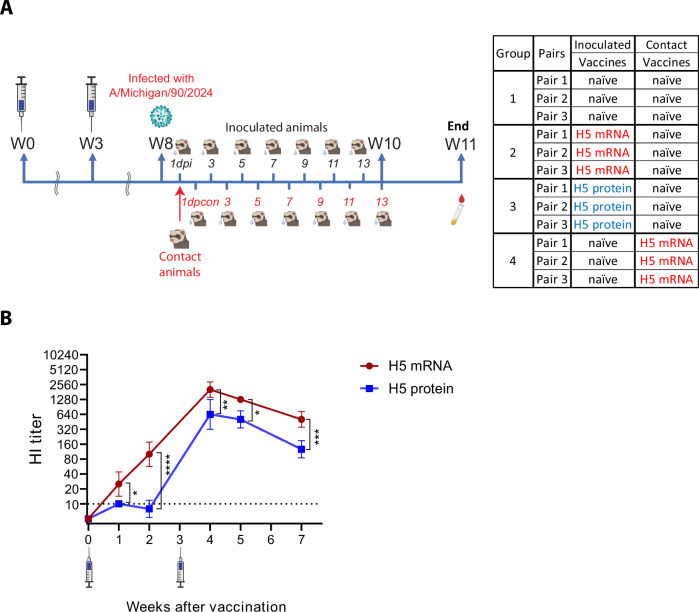
Transmission study of HPAI A(H5N1) virus in the ferret model. **A** Ferrets were vaccinated intramuscularly twice, three weeks apart, with 30 μg of mRNA vaccine encoding HA from Ast/20 (6 ferrets) or 30 μg of recombinant Ast/20 H5 HA protein adjuvanted with AddaVax (3 ferrets) as shown in the table. Naïve animals did not receive vaccines. Five weeks after the second vaccination, 3 of the H5 mRNA-vaccinated ferrets, 3 of the adjuvanted H5 protein-vaccinated ferrets, and 6 unvaccinated naïve ferrets were challenged with 10^6^ PFU of MI/24. Twenty-four hours post-infection, naïve or H5 mRNA-vaccinated ferrets were placed in the same cage as each inoculated ferret as shown in the table. Nasal wash samples were collected on days 1, 3, 5, 7, 9, 11, and 13 post-inoculation (dpi) and post-contact (dpcon). **B** Serum samples were collected from vaccinated ferrets 1, 2, 4, 5, and 7 weeks following the first vaccination and HI titers were measured using Ast/20 and 1% HRBC. Titers are reported as geometric mean titer with geometric SD. The dashed line indicates the limit of detection (10 HI) for the assay. *P < 0.05; **P < 0.01; ***P < 0.001; ****P < 0.0001.

As clade 2.3.4.4b HPAI A(H5N1) viruses may acquire human-to-human transmissibility, we used a ferret model to evaluate whether the H5 mRNA vaccine candidate could reduce virus transmission. To investigate this, we conducted a transmission experiment using ferrets in a direct contact setting, testing various combinations of inoculated/contact pairs of naïve and vaccinated ferrets (Fig. 3A). We tested four groups: (1) both inoculated and contact ferrets were unvaccinated (naïve) (Group 1), (2) H5 mRNA-vaccinated ferrets were inoculated and co-housed with naïve ferrets following infection (Group 2), (3) adjuvanted H5 protein-vaccinated ferrets were inoculated and co-housed with naïve ferrets following infection (Group 3), and (4) inoculated naïve ferrets were co-housed with H5 mRNA-vaccinated contact ferrets following infection (Group 4). All vaccinated ferrets received two intramuscular doses, three weeks apart.

Consistent with Fig. 1B, H5 mRNA-vaccinated ferrets developed a robust antibody response, with HI titers significantly higher than protein-vaccinated animals (Fig. 3B). Five weeks after the second vaccination, ferrets assigned as inoculated animals were challenged intranasally with 10⁶ PFU of A/Michigan/90/2024 (MI/24), a clade 2.3.4.4b HPAI A(H5N1) virus shown to transmit efficiently (100%) in a direct contact setting among cohoused ferrets^29,30^. Twenty-four hours post-inoculation, a contact ferret was co-housed with each inoculated ferret (three pairs per group).

**Fig. 4:**
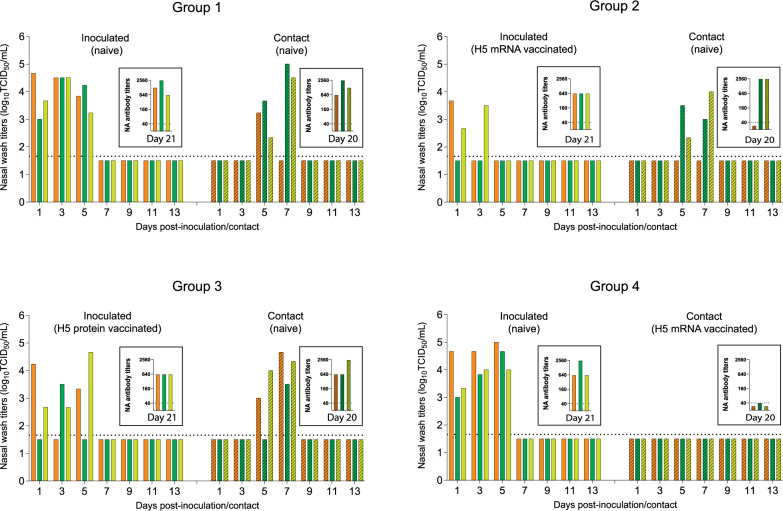
H5 mRNA vaccine candidate reduces the transmission of HPAI A(H5N1) virus between ferrets. Nasal wash samples were collected on days 1, 3, 5, 7, 9, 11, and 13 post-inoculation with MI/24 and on days 1, 3, 5, 7, 9, 11, and 13 post-contact. Viral titers were determined by TCID_50_ assays. NA antibody titers in the sera collected on day 21 post-inoculation and day 20 post-contact (11 weeks after primary vaccination) were determined by ELISA. NA antibody titers are expressed as the highest reciprocal dilution that gave an OD (450nm) above the cut-off value derived from normal ferret serum. The graphs of NA antibody titers are embedded in each graph of nasal wash titer. Each bar represents an individual ferret. The bars represent each pair of inoculated and contact ferrets in each group (open bar: inoculated animals, shaded bar: contact animals) in the same color (Pair 1: orange, Pair 2: dark green, and Pair 3: light green). Dashed lines indicate the limit of detection (1.67 log_10_TCID_50_/mL) for the assays.

To monitor transmission, NW samples were collected every other day beginning one day post-inoculation (dpi) and one day post-contact (dpcon) and infectious viral titers were determined (Fig. 4). In Group 1 (naïve inoculated/naïve contact), virus was detected in all three contact ferrets by 5 dpcon, indicating a 100% transmission rate. In Group 2 (mRNA-vaccinated inoculated/ naïve contacts), virus was detected in 2 of 3 contact ferrets (67%). Virus shedding was also limited to 2 of 3 vaccinated inoculated ferrets, and viral titers were significantly lower than those in unvaccinated inoculated animals (*P* < 0.001; Supplementary Fig. 4), likely the primary factor contributing to the reduced transmission.

It is possible that NW viral titration may miss transmission events between inoculated and contact pairs, particularly if the contact ferret rapidly cleared the infection due to limited exposure. To confirm transmission, neuraminidase (NA)-specific antibodies were measured by ELISA at week 11 (Fig. 3A). Since neither vaccine included NA, the presence of NA antibodies served as a marker of infection. In Group 2 (mRNA-vaccinated inoculated/naïve contact), although infectious virus was not detected in NW samples from all three inoculated ferrets, NA antibodies were detected in all, confirming infection. In contrast to Group 1 (naïve inoculated/naïve contact), where all contact ferrets seroconverted, NA antibodies were detected in only 2 of 3 contact ferrets in Group 2, confirming a reduced rate of transmission (Fig. 4).

In Group 3 (protein-vaccinated inoculated/naïve contacts), virus shedding from the inoculated ferrets was lower than in Group 1 at 3 dpi (*P* < 0.01), but continued through day 5, and all three contact ferrets became infected (Fig. 4). Vaccination with the adjuvanted H5 protein vaccine did not prevent transmission to any of the contact ferrets in Group 3. In Group 4 (naïve inoculated/mRNA-vaccinated contacts), transmission was completely blocked. No virus was detected in NW samples, and no NA antibodies were detected in any contact ferrets (Fig. 4).

Collectively, these results show that H5 mRNA vaccination significantly reduced viral shedding and limited transmission from vaccinated, infected ferrets. Importantly, mRNA-vaccinated contact ferrets were fully protected from infection despite direct exposure to shedding, unvaccinated animals.

### H5 mRNA vaccine candidate elicited cross-neutralizing antibody responses to recent clades 2.3.4.4b and 2.3.2.1e human viruses

**Fig. 5:**
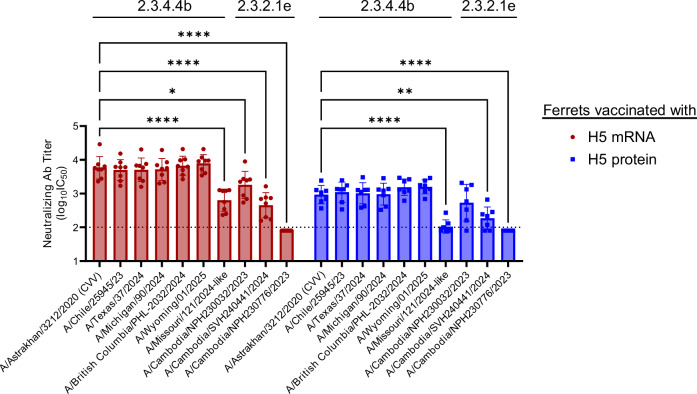
Neutralizing activity of sera from vaccinated ferrets against recent A(H5N1) human isolates. Neutralizing antibody (Ab) titers were measured in serum samples collected from ferrets one week after the second vaccination, against clades 2.3.4.4b and 2.3.2.1e HPAI A(H5) viruses isolated from humans or created by reverse genetics. Titers were determined using a focus reduction neutralization assay. Data are presented as geometric means with geometric standard deviations (SD). Statistical analysis was performed on log-transformed titers using two-way ANOVA, followed by pairwise *t*-tests with Tukey-Kramer correction for multiple comparisons. Dashed lines indicate the assay’s limit of detection (LOD; neutralizing titer of 100). Samples below the LOD were assigned a value of 80 for geometric mean calculation and statistical testing. Significance comparisons between the A/Astrakhan/3212/2020 reference strain and other isolates are indicated as *P < 0.05; **P < 0.01; ****P < 0.0001.

Human cases of clade 2.3.4.4b HPAI A(H5N1) virus infections have been reported sporadically, including 70 cases in the United States since April 1, 2024. In the Mekong Delta region of Asia, clade 2.3.2.1e HPAI A(H5N1) viruses continue to circulate among poultry and have occasionally caused human infections including in 2025^31^. To evaluate cross-neutralizing activity, ferret sera collected one week after the second vaccination (see Fig. 1B) were tested against clade 2.3.4.4b and 2.3.2.1e HPAI A(H5) viruses using a focus reduction assay (Fig. 5).

Sera from H5 mRNA-vaccinated ferrets efficiently neutralized nearly all clade 2.3.4.4b HPAI A(H5) viruses, with titers comparable to those observed against the reference candidate vaccine virus, Ast/20. Neutralization of an A/Missouri/121/2024-like virus was reduced 9-fold, likely due to the HA A156T substitution identified in viral RNA from a clinical specimen. This virus was generated by reverse genetics, as isolation of live virus was unsuccessful^32^. Sera from H5 mRNA-vaccinated ferrets showed variable neutralization against clade 2.3.2.1e HPAI A(H5N1) viruses, with no detectable activity against one strain. This likely reflects over 40 amino acid differences in HA between Ast/20 and clade 2.3.2.1e viruses, as well as strain-specific substitutions (Supplementary Tables 1–3). Sera from protein-vaccinated ferrets showed a similar neutralization pattern, although overall titers were lower than those induced by H5 mRNA vaccination (Fig. 5).

## Discussion

A Phase I clinical trial evaluating this A(H5) mRNA vaccine in humans is being conducted (ClinicalTrials.gov ID: NCT06179446) and will provide key data on its safety, immunogenicity, and other critical parameters. However, given the low incidence of A(H5N1) virus infections in humans and the risks associated with human challenge studies, clinical trials cannot directly assess protection against infection or transmission. Preclinical studies in ferrets, a gold-standard animal model for influenza, remain essential to bridge this gap^33,34^.

In this study, we conducted two independent experiments to evaluate the protective effects of an A(H5) mRNA vaccine candidate in ferrets challenged with clade 2.3.4.4b HPAI A(H5N1) viruses isolated from humans. In the first experiment, ferrets were challenged with a 2023 South American A(H5N1) isolate (A/Chile/25945/2023), and vaccination protected them from lethal disease, significantly reduced viral loads in tissues and nasal washes, and minimized clinical signs and lung pathology. In the second experiment, conducted after the 2024 U.S. dairy cattle outbreaks^35^, vaccinated ferrets were challenged with a more recent isolate (A/Michigan/90/2024) from a farm worker exposed to infected cattle. Vaccination reduced viral shedding and limited transmission to naïve contact ferrets; vaccinated contact animals were fully protected from infection, showing no viral replication or seroconversion after direct exposure to shedding, unvaccinated ferrets.

The inclusion of two different 2.3.4.4b HPAI A(H5N1) viruses to generate timely data against newly emerging strains, along with the need to include a protein vaccine control, limited the number of ferrets available per experiment. The need to conduct our studies under Animal Biosafety Level 3 (ABSL-3) containment further constrained our ability to expand sample sizes, reducing the statistical power of some findings, particularly in the transmission study (Fig. 4). Nonetheless, consistent findings from this and prior work confirm that the MI/24 virus transmits efficiently among cohoused naïve ferrets^30^. Our data in this study suggest that decreased virus shedding in H5 mRNA-vaccinated ferrets reduced transmission to cohoused contacts, but the adjuvanted H5 protein vaccine did not sufficiently reduce virus shedding to impact the rate of transmission. Interestingly, complete protection from infection was observed in H5 mRNA-vaccinated animals when exposed to virus shed by cohoused infected animals, but vaccination did not confer complete protection from virus infection and replication in vaccinated ferrets when a high dose of virus (10^6^ PFU) was administered intranasally; this may be due to dose differences or the route of inoculation. Although the precise exposure dose to the contact ferrets was unknown, viral titers measured in nasal wash samples from inoculated animals in Group 4 (peak 10^4^-10^5^ TCID_50_/mL) suggest the exposure dose was substantially lower than the intranasal inoculating dose. Of note, these data alone do not indicate that the mRNA vaccine is more effective than the protein vaccine in reducing transmission during an H5 pandemic.

Previous studies have examined the impact of vaccination on influenza A virus transmission in humans^36–39^, mice^40,41^, ferrets^42–46^, pigs^47–49^, and guinea pigs^50–53^. However, none of these studies have assessed the impact of mRNA vaccines on transmission, nor have any involved A(H5N1) viruses. More recently, a few studies have shown that mRNA vaccines provided protection against lethal challenge with clade 2.3.4.4b HPAI A(H5N1) viruses in animal models. Specifically, Hatta et al. and Furey et al. reported mRNA vaccine-mediated protection in ferrets following exposure to clade 2.3.4.4b HPAI A(H5N1) viruses isolated from birds, while Chiba et al. and Hawman et al. reported similar protective effects in mRNA-vaccinated mice challenged with dairy cattle isolates^27,54–56^. However, none of these studies assessed virus transmission. To our knowledge, this is the first demonstration that an mRNA vaccine can reduce transmission of any influenza virus and the first evidence that any vaccine can limit A(H5N1) virus transmission. The direct-contact transmission ferret model is appropriate for this vaccine study, as clade 2.3.4.4b A(H5N1) viruses are only partially transmissible in the respiratory-droplet transmission model, which could yield more variable vaccine effects and require larger animal numbers.

Despite its strengths, this study was limited by the lack of data on vaccine performance against antigenically drifted clade 2.3.4.4b HPAI A(H5N1) viruses in animals, as significant antigenic variation among recently circulating strains has rarely been observed. The mRNA vaccine, based on the 2020 A(H5N8) isolate, A/Astrakhan/3212/2020, efficiently neutralized nearly all tested clade 2.3.4.4b viruses except for the A/Missouri/121/2024-like virus, which harbored an additional N-linked glycosylation in the HA 150-loop that likely impacted antibody binding. Additionally, sera from H5 mRNA-vaccinated ferrets elicited cross-neutralization titers to genetically divergent clade 2.3.2.1e HPAI A(H5N1) viruses. While not all clade 2.3.2.1e HPAI A(H5N1) viruses were efficiently neutralized and titers were lower than those observed against clade 2.3.4.4b HPAI A(H5N1) viruses, the ability of the Ast/20 mRNA vaccine candidate to elicit some degree of cross-neutralization against a genetically divergent clade is encouraging. Further evaluation of the H5 mRNA vaccine against emerging antigenically drifted clade 2.3.4.4b variants and A(H5) viruses from other clades in animal models will be essential to define its cross-protective efficacy. Regardless of the outcomes of these studies, the adaptability of mRNA technology enables rapid vaccine updates in response to emerging antigenic changes within and across clades of A(H5N1) viruses.

In summary, this study demonstrates that an H5 mRNA vaccine provides robust protection against severe disease, reduces viral replication, and limits transmission of clade 2.3.4.4b HPAI A(H5N1) viruses. Its ability to reduce transmission in the ferret model has important implications for pandemic preparedness, as it could help slow the spread of newly emerged influenza A viruses in human populations, an essential component of outbreak control. While these preclinical results strongly suggest that the vaccine offers robust protection in ferrets, they do not address potential safety concerns, nor do they reveal adverse events which could arise in humans. As clinical trials progress, further evaluation of the safety and effectiveness of H5 mRNA vaccines in humans is imperative; such research will be critical to establishing their role in addressing the ongoing global threat posed by influenza A(H5N1).

## Methods

### Study design

This preclinical study evaluated the protective effects of an influenza mRNA vaccine against clade 2.3.4.4b highly pathogenic avian influenza (HPAI) A(H5N1) viruses. Ferrets were chosen as the animal model due to their relevance for studying influenza virus pathogenicity, transmission, and vaccine efficacy. Two in vivo experiments were conducted. In both, ferrets were immunized with either an mRNA vaccine candidate, a recombinant protein control, or phosphate-buffered saline (PBS). The first experiment assessed vaccine-mediated protection against severe disease using a lethal dose of a clade 2.3.4.4b virus isolate from Chile. Clinical signs (body weight), nasal viral shedding, and viral loads in tissues were monitored. Each group consisted of eight animals: four were monitored for clinical outcomes and nasal shedding over 14 days, and four were euthanized at 5 days post-infection for tissue viral load quantification and histopathological analysis. The second experiment evaluated the effect of vaccination on viral transmission. Ferrets were infected with a clade 2.3.4.4b human isolate from the United States (associated with the second confirmed U.S. human case in 2024). Virus shedding and direct-contact transmission were assessed. Each transmission condition included three donor-contact pairs (six animals per group), as illustrated in Fig. 3. Different virus strains were selected for each experiment to suit their respective goals: a lethal isolate for evaluating disease severity and a more transmissible isolate for assessing transmission potential.

Group sizes were selected to balance statistical power with logistical feasibility in a high-containment laboratory setting. Animals were randomly assigned to experimental groups. Blinding was not applied to the animal studies. All cell culture and serological assays followed CDC standard operating procedures, with appropriate technical replicates and controls to ensure statistical robustness.

### Biosafety and animal welfare

All research involving HPAI A(H5N1) viruses was conducted within Biosafety Level 3 enhanced (BSL-3E) or Animal Biosafety Level 3 (ABSL-3) facilities at the CDC. All personnel engaged in virus-related activities underwent comprehensive training in relevant safety protocols and specific procedural techniques. Their competence to perform tasks within BSL-3E/ABSL-3 laboratories was rigorously assessed. All ferret experiments were conducted with the approval of the CDC’s Institutional Animal Care and Use Committee (IACUC) and were conducted in an Association for Assessment and Accreditation of Laboratory Animal Care International-accredited ABSL-3 facility with enhancements as required by the U.S. Department of Agriculture^57^. The principle of using the minimum number of animals required to achieve scientific validity was strictly adhered to^58^. Procedures designed to minimize animal discomfort and pain were implemented. A clinical scoring system was used to assess the euthanasia endpoints as follows: 2 points each, hunched back/huddling or ruffled coat/piloerection/hair loss; 3 points each, dehydration, or fluctuation of body temperature; 5 points each, abnormal breathing (dyspnea, tachypnea, rales/audible breaths) anemia; 10 points each, paralysis/torticollis/moribund behavior/unresponsive, weight loss reaching 20% of baseline, reduced body temperature for greater than 3 days, frank hemorrhage/bleeding, unrelated trauma where continued exposure to cage mates is likely to lead to death, or other immediate veterinary concerns. If the clinical scoring scale reached 10 total points or above, the animals were humanely euthanized.

### Cells and viruses

Human embryonic kidney 293T (HEK-293T) cells (CRL-3216, ATCC) were maintained in Dulbecco’s Modified Eagle Medium (DMEM) supplemented with 10% fetal bovine serum. Madin-Darby canine kidney (MDCK) cells (ATCC, CCL-34) were cultured in Minimum Essential Medium (MEM) with 5% fetal bovine serum. The cells were cultured at 37 °C with 5% CO_2_. HPAI viruses A/Chile/25945/2023 (H5N1, clade 2.3.4.4b, genotype B3.2, Chile/23), A/Michigan/90/2024 (H5N1, clade 2.3.4.4b, genotype B3.13, MI/24), A/Missouri/121/2024-like (H5N1, clade 2.3.4.4b), A/British Columbia/PHL-2032/2024 (H5N1, clade 2.3.4.4b, genotype D.1.1), A/Wyoming/01/2025 (H5N1, clade 2.3.4.4b, genotype D.1.1), A/Cambodia/NPH230032/2023 (H5N1, clade 2.3.2.1e), A/Cambodia/NPH230776/2023 (H5N1, clade 2.3.2.1e), A/Cambodia/SVH240441/2024 (H5N1, clade 2.3.2.1e) and the candidate vaccine virus (CVV) of A/Astrakhan/3212/2020 (H5N8, clade 2.3.4.4b, Ast/20) were propagated in MDCK cells and titrated by plaque assay or 50% tissue culture infection dose (TCID_50_) assay with standard procedures^59,60^. The virus stocks were sequenced to confirm the absence of unwanted mutations and tested for exclusivity to rule out the presence of other subtypes of influenza virus.

### Vaccine preparation

Codon-optimized mRNA encoding a full-length HA protein from Ast/20 virus with the polybasic amino acids at HA cleavage site mutated from “REKRRKR” to “RETR” (GenBank, UJS29065) was in vitro transcribed with modified nucleosides as previously described^61^. Following in-vitro transcription, the mRNA was formulated into lipid nanoparticles using an ethanolic lipid mixture of ionizable cationic lipid as previously described^61^. Briefly, the lipid nanoparticle contains mRNA, an ionizable lipid, ((4-hydroxybutyl) azanediyl)bis(hexane-6, 1-diyl)bis(2-hexyldecanoate)), a PEGylated lipid, 2-((polyethylene glycol)-2000]-N,N-ditetradecylacetamide, a phospholipid (1,2-distearoyl-sn-glycero-3-phosphocholine (DSPC]) and a sterol lipid (cholesterol). The expression of HA protein from the mRNA vaccine was examined in cells transfected with LNP-formulated H5 mRNA by flow cytometry. H5 mRNA vaccine was 2-fold serially diluted and added to an HEK-293T cell monolayer in 12 well plates (4.0 × 10^5^ cells/well and 0.97 to 500 ng of mRNA/well). HA protein expression was measured using the broadly reactive, stem-specific anti-HA human monoclonal antibody FI6 (Creative Biolabs). The percentage of live cells expressing the HA protein (% HA+ cells) was enumerated by quantifying the number of cells that had a positive signal for bound anti-HA antibody.

The recombinant H5 protein ectodomain of Ast/20 HA was codon-optimized for expression in insect cells. The protein was expressed as trimers with a C-terminal T4 Foldon motif and His-tag in a baculovirus expression system as described previously^62–64^. The secreted recombinant HA protein was recovered from the cell culture supernatant by tangential flow filtration through a 30 kDa molecular weight cut-off membrane, followed by metal affinity chromatography and size exclusion chromatography (SEC). Stock concentration of recombinant H5 HA protein was diluted in PBS and mixed with AddaVax adjuvant (InvivoGen) to achieve the desired dose for vaccine administration in animals.

### In vitro expression of HA from the H5 mRNA vaccine

H5 mRNA was diluted in Opti-MEM (Thermo Fisher Scientific) and added to an HEK-293T cell monolayer at increasing RNA dose levels (0.97 to 500 ng/well). Opti-MEM media alone was used as a negative control. Protein expression was measured with a flow cytometer (BD FACS Fortessa) using the broadly reactive, stem-specific anti-HA human monoclonal antibody FI6 (Creative Biolabs) followed by a secondary anti-rabbit antibody conjugated to Alexa-Fluor 488 (Thermo Fisher Scientific). The percentage of cells expressing the H5 HA protein was enumerated by quantifying the number of live cells that had a positive signal for bound anti-HA antibody.

### Ferret vaccination and challenge

Male ferrets (Triple F Farms), which were confirmed serologically negative to circulating seasonal influenza A and B viruses, aged 7–12 months, were used in this study. The animals were housed in Duo-Flow Mobile units (Lab Products, LLC) throughout the experiments. Thirty µg of H5 mRNA vaccine was diluted in physiological saline to a final volume of 300 µL. Recombinant H5 HA protein was diluted in PBS and mixed at a 1:1 ratio with AddaVax adjuvant (InvivoGen) to a final volume of 300 µL. All animals were vaccinated with 300 µL of vaccine or PBS intramuscularly in a thigh. Serum samples were collected at the time points indicated in Figs. 1A, 3A, from the cranial vena cava of ferrets while under anesthesia (ketamine/xylazine).

For the challenge experiment, vaccinated ferrets were anesthetized and intranasally inoculated with 10^4^ TCID_50_ of the Chile/23 HPAI A(H5N1) virus at 5 weeks post-second vaccination. Clinical signs of disease, body weight, and body temperature were monitored daily for 14 days. Nasal wash (NW) samples were collected from all remaining animals in each group on days 2, 4, 6, 8, and 10 post-challenge. Organ samples (lung, trachea, nasal turbinates, spleen, liver, kidney, small intestine, large intestines, brain tissues, and olfactory bulb) were collected for virus titration and histopathological analysis on day 5 post-challenge. NW samples and organ samples were titrated by TCID_50_ assay on MDCK cells as described previously^27^.

### Direct contact transmission model

Male ferrets (Triple F Farms) were intranasally administered 10^6^ PFU of MI/24 A(H5N1) virus in 1 mL PBS (0.5 mL per nostril) on week 8, 5 weeks following the second vaccination. To establish contact, at 24 hours post-inoculation, an uninfected ferret (naïve or vaccinated depending on group) was placed in the same cage as each inoculated ferret. Four separate groups of ferrets (three ferret pairs per group) were established to assess the effect of vaccination on infection, virus shedding, and transmission (Fig. 3A): Group 1. Naïve inoculated ferrets housed with naïve contacts. Group 2. H5 mRNA-vaccinated inoculated animals housed with naïve contacts. Group 3. Adjuvanted H5 protein-vaccinated inoculated ferrets housed with naïve contacts. Group 4. Naïve inoculated ferrets were housed with H5 mRNA-vaccinated contacts.

NW samples were collected on days 1, 3, 5, 7, 9, 11, and 13 post-inoculation and post-contact by sedating the ferrets (ketamine/xylazine) followed by rinsing the nares with 1 mL PBS (0.5 mL each side) containing penicillin/streptomycin, gentamicin, and 0.1% bovine serum albumin (BSA). NW samples were titrated by TCID_50_ assay on MDCK cells. Convalescent serum was obtained by collecting 1 mL of blood from the cranial vena cava on days 22 or 21 post-inoculation/contact, respectively. Whole blood was transferred to an SST tube (Becton Dickinson), centrifuged per the manufacturer instructions, transferred to a cryotube and stored at −80 °C until assays could be conducted.

### Hemagglutination Inhibition (HI) Assay

HI assays were performed against the candidate vaccine virus (CVV) of Ast/20 or MI/24 virus with 1% horse red blood cells (HRBCs). Briefly, ferret serum samples were treated with receptor-destroying enzyme (RDE; Denka Seiken Co., Ltd) at 37 °C for 16 to 20 hours followed by inactivation at 56 °C for 30 min. After adsorption of non-specific agglutinins with HRBCs, the RDE-treated serum samples were serially diluted 2-fold with PBS and mixed with eight hemagglutination units of virus, followed by incubation at room temperature for 30 min. 1% HRBCs were then added to the mixtures and further incubated at room temperature for 60 min. HI titers are expressed as the highest reciprocal dilution that resulted in inhibition of agglutination.

### ELISA

ELISA was performed as described previously with slight modification^65,66^. Recombinant monomer NA protein of clade 2.3.4.4b HPAI A(H5N1) virus, A/American wigeon/South Carolina/22-000345-001/2021, was obtained from Sino Biological. 96-well ELISA plates were coated with NA protein overnight at 4 °C. The plates were blocked with 1% BSA in PBS and then were incubated with four-fold serial diluted ferret sera. Anti-ferret IgG Primary Ab (ImmunoSpot) and a horseradish peroxidase (HRP)-conjugated goat anti-mouse IgG (MilliporeSigma) were used as the secondary and tertiary antibody, respectively. The signal was developed using 3,3’,5,5’-tetramethylbenzidine (TMB) (MilliporeSigma) as the substrate and stopped with TMB Stop Solution (SeraCare). The plates were read at a wavelength of 450 nm. The antibody titers are expressed as the highest reciprocal dilution that gave an OD above the cut-off value derived from normal ferret serum.

### Quantification of viral RNA in ferret organ samples by digital PCR (dPCR)

dPCR for the quantification of viral RNAs in ferret organ samples was performed as described previously^27^. Briefly, RNA was extracted from organ homogenate in MagNA Pure LC RNA isolation tissue lysis buffer (Roche) with a MagNA Pure Cellular RNA Large Volume Kit (Roche) on the MagNA Pure 96 system (Roche). For absolute RNA quantification, the QIAcuity digital PCR System (Qiagen) was used. Reactions were prepared using the QIAcuity OneStep Advanced Probe Kit with InfA primer/probes from CDC rRT-PCR Flu Panel: Influenza A/B Typing Kit (VER 2; 510 (k) number K200370). Each RNA sample was loaded on QIAcuity 8.5k x 96-well plates with default parameters for priming and cycling conditions. Raw copy/µL numbers for the reaction mixture were converted to copy/mg tissue. A stock virus with a known titer was used as a positive control, while a buffer-only negative control was included during RNA extraction and carried through the quantification process.

### Pathological analyses

Ferret tissues were fixed in 10% neutral buffered formalin and processed as previously described^27^. Hematoxylin and eosin (H&E) staining and immunohistochemistry were performed by the Department of Pathology, College of Veterinary Medicine, University of Georgia. For immunohistochemistry, sample slides were pre-treated with citrate and pressure cooked at 110 °C for 15 min. They were then blocked with 3% hydrogen peroxide for 20 min and then Power Block for 5 min. The slides were incubated with goat polyclonal influenza A virus antibody (ab20841, Abcam) for 1 hour, with biotinylated rabbit anti-goat IgG secondary antibody (BA-5000-1.5, Vector Laboratories) for 10 min, and then with 4plus Streptavidin AP Label (AP605, Biocare Medical) for 10 min. Warp Red (Biocare Medical) was used as chromogen. The slides were evaluated qualitatively in a blinded manner by light microscopy by a board-certified veterinary pathologist using a Zeiss Axio Imager M2m microscope. Slides were scanned using a Leica Aperio AT2 digital pathology slide scanner, and photomicrographs were captured from whole slide images using Aperio ImageScope (v12.3.2.8013).

### Focus Reduction Assay

The focus reduction assay was performed as previously described^27^. Briefly, 2-fold serially diluted antiserum samples and 600 focus-forming units of virus were added to 96-well plates containing confluent MDCK cells and incubated at 37 °C for 2 hours. An equal volume of overlay, consisting of 1.2% Avicel RC/CL in 2X MEM with 1 μg/mL TPCK-treated trypsin, 0.1% BSA fraction V, and antibiotics, was then added. After 14 hours incubation, the cells were fixed with 70% cold ethanol at 4 °C for 30 min. After fixation, the fluorescent NA substrate (BTP3-Neu5Ac Na) was added to washed reaction plates to visualize the Foci quantity, replacing the immunostaining. The values of 50% neutralization (NT_50_) were calculated in GraphPad Prism 10.3.

### Statistical analyses

Statistical analyses of HI titers and body weight changes were performed using two-way analysis of variance (ANOVA), with timepoint and treatment group as independent variables. Log-transformed HI titers were analyzed to compare responses across vaccine groups over time. For post hoc comparisons following ANOVA, Šídák’s multiple comparisons test was applied. For bar graph data, pairwise *t*-tests with Tukey-Kramer’s adjustment for multiple comparisons were conducted on log-transformed viral titers and neutralizing antibody titers. Animal survival was analyzed using the log-rank (Mantel-Cox) test. All analyses were performed using GraphPad Prism version 10.3, and *p* values < 0.05 were considered statistically significant.

## Supplementary information

Supplementary Information

## Data Availability

All data associated with this study are in the paper or the Supplementary Materials. Requests for viruses and serum samples should be directed to the CDC, whereas requests for mRNA-LNP should be directed to Pfizer. Please note that material transfer agreements or other applicable legal agreements may be required for materials sharing.

## Code availability

No custom code or scripts were used to generate or analyze datasets. Full details of the statistical methods used for figure generation are provided in “Statistical Analyses”. Sequence alignments were conducted with DNAstar Lasergene MegAlign Pro version 17 and all other analyses were conducted with GraphPad Prism version 10.3.

## References

[1] World Health Organization. *Cumulative number of confirmed human cases for avian influenza A(H5N1) reported to WHO, 2003-2025, 19 March 2025*, www.who.int/publications/m/item/cumulative-number-of-confirmed-human-cases-for-avian-influenza-a(h5n1)-reported-to-who--2003-2025--19-march-2025 (WHO, 2025).

[2] Webby, R. J. & Uyeki, T. M. An update on highly pathogenic avian influenza A(H5N1) Virus, Clade 2.3.4.4b. *J. Infect. Dis.* **230**, 533–542 (2024). 39283944 10.1093/infdis/jiae379

[3] Bevins, S. N. et al. Intercontinental movement of highly pathogenic avian influenza A(H5N1) Clade 2.3.4.4 virus to the United States, 2021. *Emerg. Infect. Dis.* **28**, 1006–1011 (2022). 35302933 10.3201/eid2805.220318 PMC9045435

[4] Miller, B. J. Why unprecedented bird flu outbreaks sweeping the world are concerning scientists. *Nature* **606**, 18–19 (2022). 35618804 10.1038/d41586-022-01338-2

[5] Chen, H. et al. Properties and dissemination of H5N1 viruses isolated during an influenza outbreak in migratory waterfowl in western China. *J. Virol.* **80**, 5976–5983 (2006). 16731936 10.1128/JVI.00110-06 PMC1472608

[6] Stokstad, E. Deadly flu spreads through North American birds. *Science* **376**, 441–442 (2022). 35482856 10.1126/science.abq7228

[7] Nagy, A., Cernikova, L. & Stara, M. A new clade 2.3.4.4b H5N1 highly pathogenic avian influenza genotype detected in Europe in 2021. *Arch. Virol.* **167**, 1455–1459 (2022). 35469095 10.1007/s00705-022-05442-6

[8] Liang, Y. et al. Novel Clade 2.3.4.4b Highly pathogenic avian influenza A H5N8 and H5N5 Viruses in Denmark, 2020. *Viruses* **13**, 10.3390/v13050886 (2021). 10.3390/v13050886 PMC8151437 34065033

[9] Caliendo, V. et al. Transatlantic spread of highly pathogenic avian influenza H5N1 by wild birds from Europe to North America in 2021. *Sci. Rep.* **12**, 11729 (2022). 35821511 10.1038/s41598-022-13447-z PMC9276711

[10] Youk, S. et al. H5N1 highly pathogenic avian influenza clade 2.3.4.4b in wild and domestic birds: Introductions into the United States and reassortments, December 2021-April 2022. *Virology* **587**, 109860 (2023). 37572517 10.1016/j.virol.2023.109860

[11] U.S. Department of Agriculture. *Detections of Highly Pathogenic Avian Influenza in Mammals*, www.aphis.usda.gov/livestock-poultry-disease/avian/avian-influenza/hpai-detections/mammals (2024).

[12] Peacock, T. P. et al. The global H5N1 influenza panzootic in mammals. *Nature*. 10.1038/s41586-024-08054-z (2024). 10.1038/s41586-024-08054-z 39317240

[13] Jimenez-Bluhm, P. et al. Detection and phylogenetic analysis of highly pathogenic A/H5N1 avian influenza clade 2.3.4.4b virus in Chile, 2022. *Emerg. Microbes Infect.* **12**, 2220569 (2023). 37254689 10.1080/22221751.2023.2220569 PMC10283444

[14] Ariyama, N. et al. Highly pathogenic avian influenza A(H5N1) Clade 2.3.4.4b virus in wild birds, Chile. *Emerg. Infect. Dis.* **29**, 1842–1845 (2023). 37487166 10.3201/eid2909.230067 PMC10461661

[15] Ulloa, M. et al. Mass mortality event in South American sea lions (Otaria flavescens) correlated to highly pathogenic avian influenza (HPAI) H5N1 outbreak in Chile. *Vet. Q* **43**, 1–10 (2023). 37768676 10.1080/01652176.2023.2265173 PMC10588531

[16] World Health Organization. *Human infection caused by Avian Influenza A (H5) - Chile*, www.who.int/emergencies/disease-outbreak-news/item/2023-DON453 (WHO, 2023).

[17] Castillo, A. et al. The first case of human infection with H5N1 avian Influenza A virus in Chile. *J. Travel Med.* **30**, 10.1093/jtm/taad083 (2023). 10.1093/jtm/taad083 PMC10481412 37310882

[18] U.S. Department Agriculture. *HPAI Confirmed Cases in Livestock*, www.aphis.usda.gov/livestock-poultry-disease/avian/avian-influenza/hpai-detections/hpai-confirmed-cases-livestock (U.S. Department of Agriculture, 2025).

[19] Uyeki, T. M. et al. Highly pathogenic avian influenza A(H5N1) virus infection in a dairy farm worker. *N. Engl. J. Med.* **390**, 2028–2029 (2024). 38700506 10.1056/NEJMc2405371

[20] Texas Department of State Health Services. *First Case of Novel Influenza A (H5N1) in Texas, March 2024*, www.dshs.texas.gov/news-alerts/first-case-novel-influenza-h5n1-texas-march-2024#:~:text=Summary,patient%27s%20primary%20symptom%20was%20conjunctivitis (Texas Department of State Health Services, 2024).

[21] Centers for Disease Control and Prevention (U.S.). *Centers for Disease Control and Prevention: H5 Bird Flu: Current Situation*, www.cdc.gov/bird-flu/situation-summary/index.html (CDC, 2024).

[22] Garg, S. et al. Highly Pathogenic Avian Influenza A(H5N1) Virus Infections in Humans. *N. Engl. J. Med.* **392**, 843–854 (2025). 39740051 10.1056/NEJMoa2414610

[23] U.S. Food and Drug Administration. *Vaccines Licensed for Use in the United States*, www.fda.gov/vaccines-blood-biologics/vaccines/vaccines-licensed-use-united-states (FAO, 2025).

[24] Walsh, E. E. et al. Safety and Immunogenicity of Two RNA-Based Covid-19 Vaccine Candidates. *N. Engl. J. Med.* **383**, 2439–2450 (2020). 33053279 10.1056/NEJMoa2027906 PMC7583697

[25] Polack, F. P. et al. Safety and Efficacy of the BNT162b2 mRNA Covid-19 Vaccine. *N. Engl. J. Med.* **383**, 2603–2615 (2020). 33301246 10.1056/NEJMoa2034577 PMC7745181

[26] Hauguel, T. et al. Preclinical immunogenicity and safety of hemagglutinin-encoding modRNA influenza vaccines. *NPJ Vaccines* **9**, 183 (2024). 39375384 10.1038/s41541-024-00980-3 PMC11488230

[27] Hatta, M. et al. An influenza mRNA vaccine protects ferrets from lethal infection with highly pathogenic avian influenza A(H5N1) virus. *Sci. Transl. Med.* **16**, eads1273 (2024). 39693411 10.1126/scitranslmed.ads1273 PMC12100637

[28] Stephenson, I., Wood, J. M., Nicholson, K. G. & Zambon, M. C. Sialic acid receptor specificity on erythrocytes affects detection of antibody to avian influenza haemagglutinin. *J. Med Virol.* **70**, 391–398 (2003). 12767002 10.1002/jmv.10408

[29] Morse, J. et al. Influenza A(H5N1) Virus Infection in Two Dairy Farm Workers in Michigan. *N. Engl. J. Med.* **391**, 963–964 (2024). 39115081 10.1056/NEJMc2407264

[30] Brock, N. et al. Avian Influenza A(H5N1) Isolated from Dairy Farm Worker, Michigan. *Emerg. Infect. Dis.* **31**, 1253–1256 (2025). 40314761 10.3201/eid3106.250386 PMC12123903

[31] World Health Organization. *Influenza at the human-animal interface summary and assessment, 19 March 2025*, www.who.int/publications/m/item/influenza-at-the-human-animal-interface-summary-and-assessment-19-march-2025 (WHO, 2025).

[32] Wang, L. et al. Impact of naturally occurring hemagglutinin substitutions on antigenicity and fitness of influenza A(H5N1) virus. *npj Viruses* **3**, 72 (2025). 41034380 10.1038/s44298-025-00154-5 PMC12489106

[33] Belser, J. A. et al. A guide for the use of the ferret model for influenza virus infection. *Am. J. Pathol.* **190**, 11–24 (2020). 31654637 10.1016/j.ajpath.2019.09.017 PMC8264465

[34] Belser, J. A., Pulit-Penaloza, J. A. & Maines, T. R. Ferreting out influenza virus pathogenicity and transmissibility: past and future risk assessments in the ferret model. *Cold Spring Harb. Perspect. Med.* **10**, 10.1101/cshperspect.a038323 (2020). 10.1101/cshperspect.a038323 PMC7328449 31871233

[35] Centers for Disease Control and Prevention (U.S.). *Current Situation: Bird Flu in Dairy Cows*, www.cdc.gov/bird-flu/situation-summary/mammals.html (CDC, 2025).

[36] Ainslie, K. E. C., Haber, M. & Orenstein, W. A. Challenges in estimating influenza vaccine effectiveness. *Expert Rev. Vaccines* **18**, 615–628 (2019). 31116070 10.1080/14760584.2019.1622419 PMC6594904

[37] Grijalva, C. G. et al. Estimated Effectiveness of Influenza Vaccines in Preventing Secondary Infections in Households. *JAMA Netw. Open* **7**, e2446814 (2024). 39570586 10.1001/jamanetworkopen.2024.46814 PMC11582933

[38] Rolfes, M. A. et al. Household Transmission of Influenza A Viruses in 2021-2022. *JAMA* **329**, 482–489 (2023). 36701144 10.1001/jama.2023.0064 PMC9880862

[39] Tsang, T. K., Lau, L. L. H., Cauchemez, S. & Cowling, B. J. Household transmission of influenza virus. *Trends Microbiol* **24**, 123–133 (2016). 26612500 10.1016/j.tim.2015.10.012 PMC4733423

[40] Price, G. E., Lo, C. Y., Misplon, J. A. & Epstein, S. L. Reduction of influenza virus transmission from mice immunized against conserved viral antigens is influenced by route of immunization and choice of vaccine antigen. *Vaccine* **36**, 4910–4918 (2018). 30037481 10.1016/j.vaccine.2018.06.051

[41] Price, G. E., Lo, C. Y., Misplon, J. A. & Epstein, S. L. Reduction of influenza A Virus transmission in mice by a universal intranasal vaccine candidate is long-lasting and does not require antibodies. *J. Virol.* **96**, e0032022 (2022). 35638848 10.1128/jvi.00320-22 PMC9215256

[42] Septer, K. M. et al. Vaccine-induced NA immunity decreases viral shedding, but does not disrupt chains of airborne transmission for the 2009 pandemic H1N1 virus in ferrets. *mBio* **15**, e0216124 (2024). 39248566 10.1128/mbio.02161-24 PMC11481891

[43] Houser, K. V., Katz, J. M. & Tumpey, T. M. Seasonal trivalent inactivated influenza vaccine does not protect against newly emerging variants of influenza A (H3N2v) virus in ferrets. *J. Virol.* **87**, 1261–1263 (2013). 23115290 10.1128/JVI.02625-12 PMC3554089

[44] Houser, K. V., Pearce, M. B., Katz, J. M. & Tumpey, T. M. Impact of prior seasonal H3N2 influenza vaccination or infection on protection and transmission of emerging variants of influenza A(H3N2)v virus in ferrets. *J. Virol.* **87**, 13480–13489 (2013). 24089569 10.1128/JVI.02434-13 PMC3838242

[45] Nachbagauer, R. et al. Hemagglutinin stalk immunity reduces influenza virus replication and transmission in ferrets. *J. Virol.* **90**, 3268–3273 (2015). 26719251 10.1128/JVI.02481-15 PMC4810634

[46] McKay, P. F. et al. Polymer formulated self-amplifying RNA vaccine is partially protective against influenza virus infection in ferrets. *Oxf. Open Immunol.* **3**, iqac004 (2022). 35996628 10.1093/oxfimm/iqac004 PMC9384352

[47] Everett, H. E. et al. Vaccines that reduce viral shedding do not prevent transmission of H1N1 pandemic 2009 swine influenza A Virus infection to unvaccinated pigs. *J. Virol.* **95**, 10.1128/JVI.01787-20 (2021). 10.1128/JVI.01787-20 PMC7851569 33268518

[48] Romagosa, A. et al. Vaccination of influenza a virus decreases transmission rates in pigs. *Vet. Res.* **42**, 120 (2011). 22185601 10.1186/1297-9716-42-120 PMC3258204

[49] Li, C. et al. Quantifying the impact of vaccination on transmission and diversity of influenza A variants in pigs. *J. Virol.* **98**, e0124524 (2024). 39530665 10.1128/jvi.01245-24 PMC11651001

[50] Lowen, A. C. et al. Blocking interhost transmission of influenza virus by vaccination in the guinea pig model. *J. Virol.* **83**, 2803–2818 (2009). 19153237 10.1128/JVI.02424-08 PMC2655561

[51] McMahon, M. et al. Mucosal Immunity against Neuraminidase Prevents Influenza B Virus Transmission in Guinea Pigs. *mBio* **10**, 10.1128/mBio.00560-19 (2019). 10.1128/mBio.00560-19 PMC6529633 31113896

[52] Yang, W. et al. A live attenuated vaccine prevents replication and transmission of H7N9 highly pathogenic influenza viruses in mammals. *Emerg. Microbes Infect.* **7**, 153 (2018). 30206210 10.1038/s41426-018-0154-6 PMC6133968

[53] Tan, J. et al. Human Anti-neuraminidase antibodies reduce airborne transmission of clinical influenza virus isolates in the guinea pig model. *J. Virol.* **96**, e0142121 (2022). 34669506 10.1128/JVI.01421-21 PMC8791283

[54] Chiba, S. et al. Protective effects of an mRNA vaccine candidate encoding H5HA clade 2.3.4.4b against the newly emerged dairy cattle H5N1 virus. *EBioMedicine* **109**, 105408 (2024). 39481207 10.1016/j.ebiom.2024.105408 PMC11565041

[55] Furey, C. et al. Development of a nucleoside-modified mRNA vaccine against clade 2.3.4.4b H5 highly pathogenic avian influenza virus. *Nat. Commun.* **15**, 4350 (2024). 38782954 10.1038/s41467-024-48555-z PMC11116520

[56] Hawman, D. W. et al. Clade 2.3.4.4b but not historical clade 1 HA replicating RNA vaccine protects against bovine H5N1 challenge in mice. *Nat. Commun.* **16**, 655 (2025). 39809744 10.1038/s41467-024-55546-7 PMC11732985

[57] Chosewood, L. C., Wilson, D. E., Centers for Disease Control and Prevention (U.S.) & National Institutes of Health (U.S.). *Biosafety in microbiological and biomedical laboratories*. 5th edn, (U.S. Dept. of Health and Human Services, Public Health Service, Centers for Disease Control and Prevention, National Institutes of Health, 2009).

[58] Russell, W. M. S. & Burch, R. L. *The principles of humane experimental technique*. (Methuen, 1959).

[59] World Health Organization. *WHO manual on animal influenza diagnosis and surveillance*, iris.who.int/handle/10665/68026 (WHO, 2002).

[60] Balish, A. L., Katz, J. M. & Klimov, A. I. Influenza: propagation, quantification, and storage. *Curr. Protoc. Microbiol* **Chapter 15**, 15G 11 11–15G 11 24 (2013). 10.1002/9780471729259.mc15g01s29 23686827

[61] Vogel, A. B. et al. BNT162b vaccines protect rhesus macaques from SARS-CoV-2. *Nature* **592**, 283–289 (2021). 33524990 10.1038/s41586-021-03275-y

[62] Yang, H., Carney, P. & Stevens, J. Structure and Receptor binding properties of a pandemic H1N1 virus hemagglutinin. *PLoS Curr.* **2**, RRN1152 (2010). 20352039 10.1371/currents.RRN1152 PMC2846141

[63] Yang, H., Carney, P. J., Chang, J. C., Villanueva, J. M. & Stevens, J. Structural analysis of the hemagglutinin from the recent 2013 H7N9 influenza virus. *J. Virol.* **87**, 12433–12446 (2013). 24027325 10.1128/JVI.01854-13 PMC3807915

[64] Stevens, J. et al. Structure and receptor specificity of the hemagglutinin from an H5N1 influenza virus. *Science* **312**, 404–410 (2006). 16543414 10.1126/science.1124513

[65] Hossain, M. J. et al. Virus-like particle vaccine containing hemagglutinin confers protection against 2009 H1N1 pandemic influenza. *Clin. Vaccin. Immunol.* **18**, 2010–2017 (2011). 10.1128/CVI.05206-11 PMC3232700 22030367

[66] Kamal, R. P. et al. Inactivated H7 influenza virus vaccines protect mice despite inducing only low levels of neutralizing antibodies. *J. Virol.* **91**, 10.1128/JVI.01202-17 (2017). 10.1128/JVI.01202-17 PMC5625511 28768855

