# Supplementary material for: Influenza mRNA vaccine reduces pathogenicity and transmission of A(H5N1) virus in a ferret model
Source: NPJ Vaccines. 2025 Nov 29;10:263. doi: 10.1038/s41541-025-01318-3 (PMC12728207; doi:10.1038/s41541-025-01318-3)
Supplement: Supplementary file 1 — Supplementary Information [file 41541_2025_1318_MOESM1_ESM.pdf]

Supplementary Materials for

Influenza mRNA vaccine reduces pathogenicity and transmission of A(H5N1) virus in a ferret model

Masato Hatta, Nicole Brock, Teresa Hauguel, Chenchen Feng, Ying Huang, Jana M. Ritter, Yasuko Hatta, Matthew W. Keller, Ivna De Souza, Jaber Hossain, Elizabeth A. Pusch, Thomas Rowe, Herg Zhang, Liyang Cui, Sarah O'Leary, Juan A. De La Cruz, Monique C. Johnson, Jessica A. Belser, Xiangjie Sun, Jimma Liddell, Margaret Creech, Joseph R. Rouse, Paul Carney, Jessie Chang, Michael Currier, Li Wang, Marie K. Kirby, Han Di, John R. Barnes, James Stevens, Vivien G. Dugan, C. Todd Davis, David E. Wentworth, Pirada Suphaphiphat Allen, Taronna R Maines, Bin Zhou

Corresponding authors: Pirada Suphaphiphat Allen, Pirada.Allen@pfizer.com; Taronna R Maines, zay9@cdc.gov; Bin Zhou, nmb7@cdc.gov

The PDF file includes:

Supplementary Figs. 1 to 4
Supplementary Tables 1 to 3

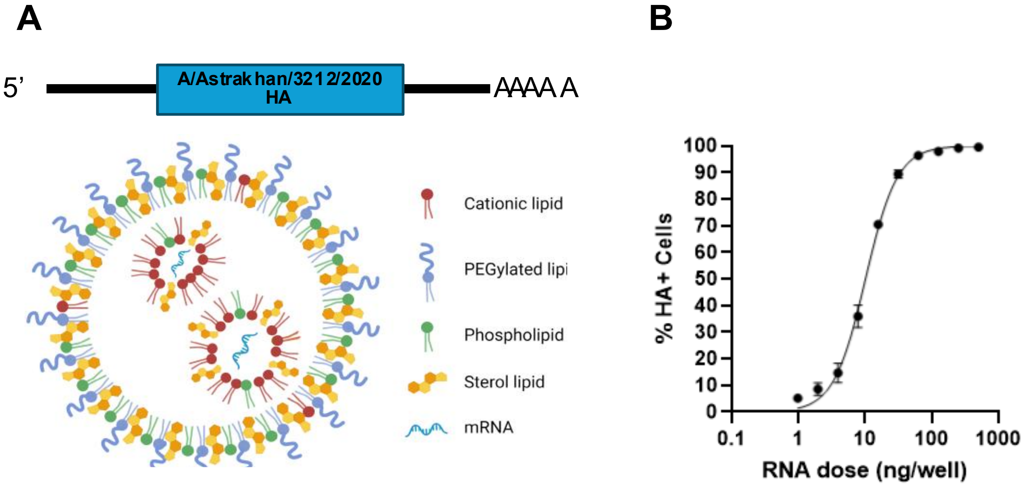

Supplementary Fig. 1. A/Astrakhan/3212/2020 lipid nanoparticle (LNP)-formulated nucleoside-modified mRNA vaccine candidate. (A) Codon-optimized mRNA encoding a full-length HA protein from Ast/20 with the polybasic amino acids at the HA cleavage site deleted, was in vitro transcribed and formulated into lipid nanoparticles containing an ionizable lipid, a PEGylated lipid, a phospholipid and a sterol lipid. **(B)** Flow cytometry measurement of HA protein expression in cells transfected with LNP-formulated H5 mRNA. LNP-formulated nucleoside-modified mRNA encoding HA from Ast/20 was serially diluted and added to an HEK-293T cell monolayer in 12-well plates. HA protein expression was detected using a broadly reactive monoclonal antibody. The percentage of live cells expressing the HA protein (HA+ cells) was enumerated by quantifying the number of cells that had a positive signal for bound anti-HA antibody. Data shown is mean with standard deviation of duplicate measurements from one representative experiment.

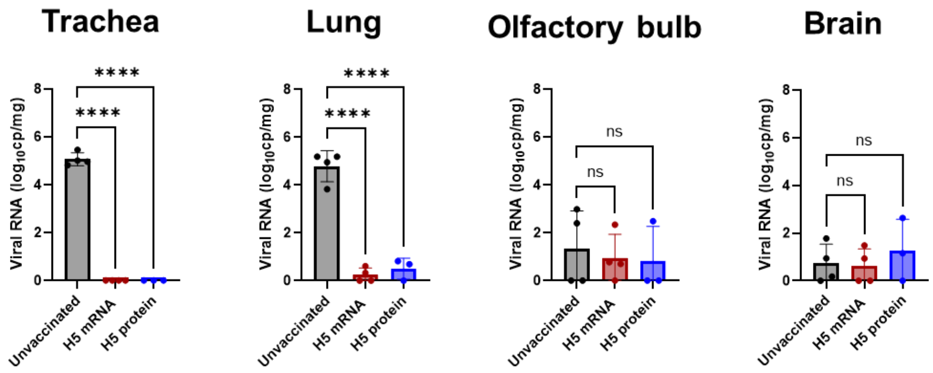

Supplementary Fig. 2. Quantification of viral RNA in organ samples by dPCR. Organ samples were collected from 4 ferrets (Group 1 and 2) and 3 ferrets (Group 3) on day 5 post-challenge and homogenized for quantification of viral RNA by dPCR. Cp/mg: copies per milligram of tissue. ****$P < 0.0001$, ns = not significant.

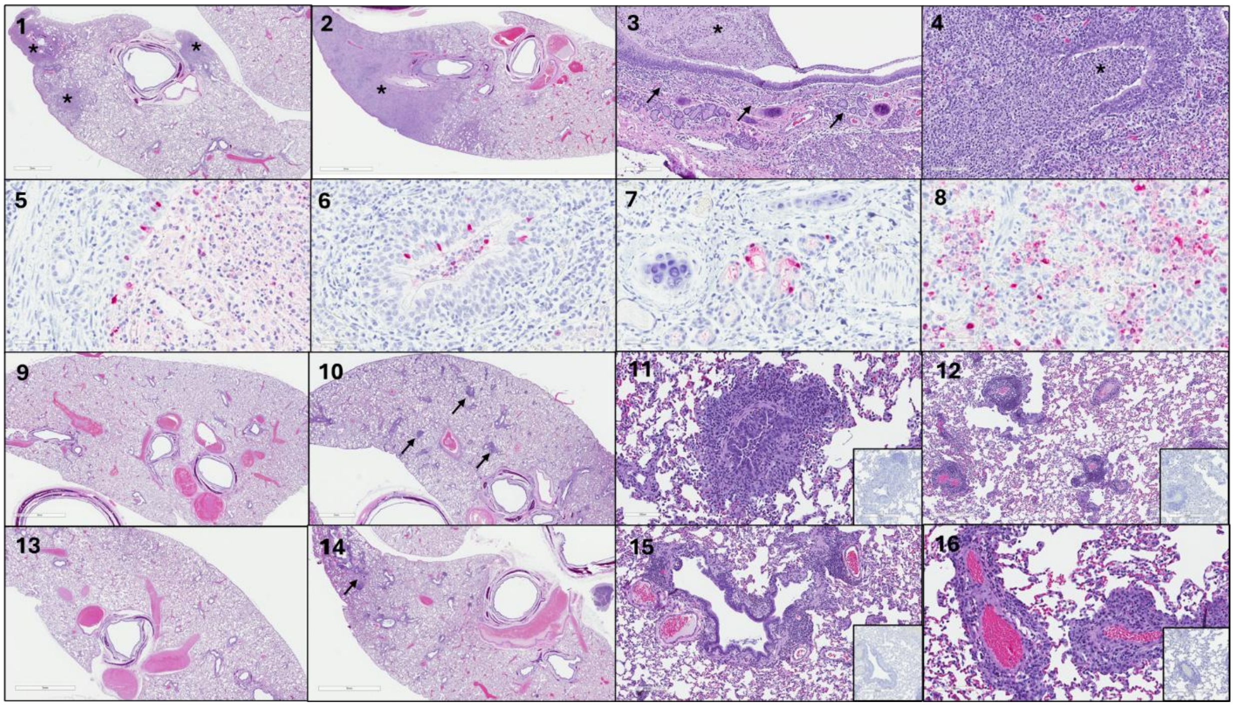

Supplementary Fig. 3. H5 mRNA vaccine candidate prevents pneumonia and is associated with an absence of detectable viral antigen in ferret lungs. Lungs were collected 5 days post-challenge with Chile/23, and viral antigen was assessed by immunohistochemistry (IHC). **(1-8) Unvaccinated ferret lungs.** (1, 2) Lungs from two different unvaccinated animals show focal to extensive regions of pneumonia (*). (3) Bronchial lumen contains neutrophils and mucus (*), and there is mononuclear inflammation in the bronchial submucosa and around glands (arrows). (4) A partially necrotic bronchiole is filled by neutrophils, which spill into the surrounding alveolar parenchyma. **(5-8)** Labeling of viral antigen by IHC in (5) bronchial epithelium and exudate, (6) bronchiolar epithelium and exudate, (7) bronchial submucosal glands, and (8) epithelial and inflammatory cells in the pneumonic lung. **(9-12) Lungs from ferrets vaccinated with H5 mRNA vaccine.** (9, 10) Lungs from two different ferrets vaccinated with H5 mRNA vaccine. There are perivascular and peribronchiolar infiltrates (arrows) in one animal, but no pneumonia in either animal. (11) Bronchiolitis, with mixed inflammation in the bronchiolar wall and lumen [inset: no immunostaining for viral antigens]. (12) Dense perivascular lymphocytic infiltrates [inset: no immunostaining for viral antigens]. **(13-16) Lungs from ferrets vaccinated with adjuvanted H5 protein vaccine.** (13, 14) Lungs from two different ferrets vaccinated with adjuvanted H5 protein vaccine. There is focal atelectasis and peribronchiolar inflammation in one animal (arrow), but no pneumonia in either animal. (15) Mild peribronchiolar lymphocytic infiltrates [inset: no immunostaining for viral antigens]. (16) Perivascular infiltrates [inset: no immunostaining for viral antigens]. Hematoxylin-eosin stain (1-4, 9-16). Immunohistochemistry for influenza virus A (5-8 and insets).

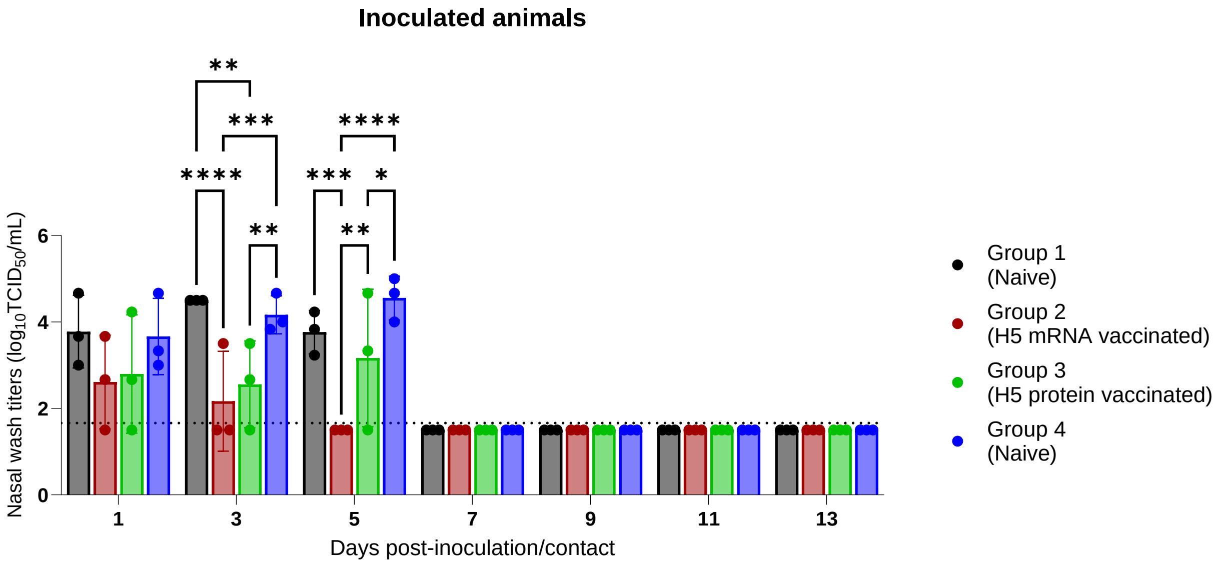

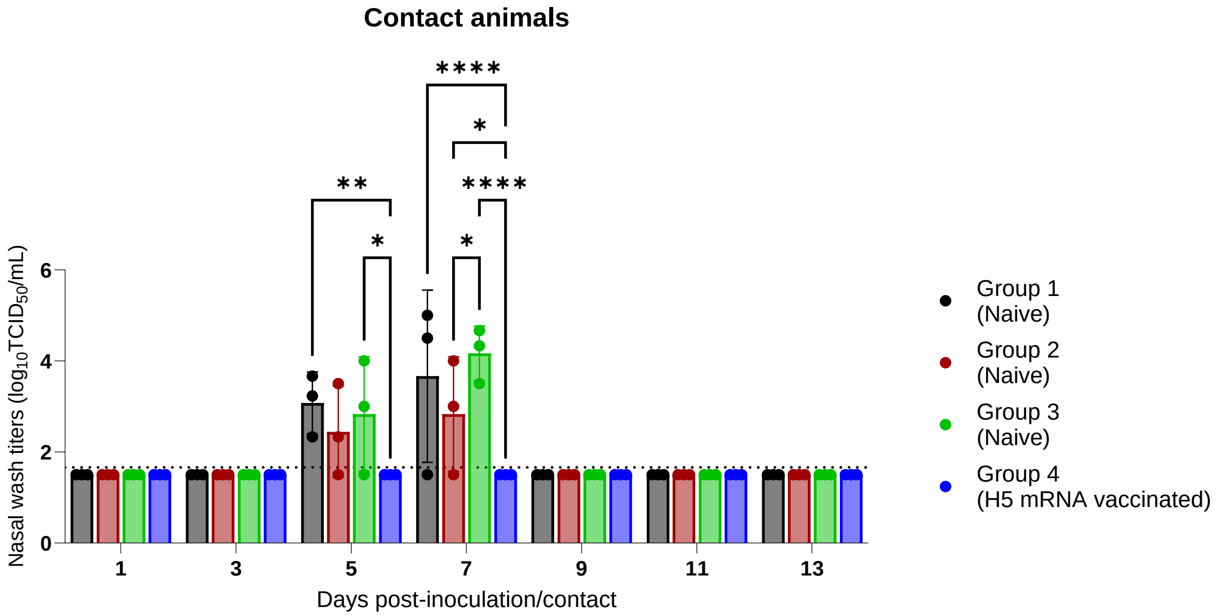

Supplementary Fig. 4. Summary and statistical analysis of viral titers in nasal wash samples in transmission experiment. The viral titers in nasal wash samples shown in Fig. 4 were summarized in each group and analyzed statistically. Data are presented as geometric mean ± SD. Viral titers were analyzed by pairwise t-test with Tukey-Kramer adjustment for multiple comparisons on the log titers. Dashed lines indicate the limit of detection (1.67 $log_{10}TCID_{50}/mL$) for the assays. $*P < 0.05$; $**P < 0.01$; $***P < 0.001$; $****P < 0.0001$.

Supplementary Table 1.

Amino acid comparison of HA proteins among clade 2.3.4.4b HPAI A(H5) human isolates used in FRA

Clade	Virus	Amino acid at position:										
		36	104	115	136	140	156	186	195	210	475	510
2.3.4.4b	A/Astrakhan/3212/2020	T	L	L	P	A	A	E	T	A	N	I
	A/Chile/25945/2023	T	M	Q	P	A	A	E	T	V	N	V
	A/Texas/37/2024	T	M	Q	P	A	A	E	I	A	N	V
	A/Michigan/90/2024	T	M	Q	P	A	A	E	I	A	N	V
	A/British Columbia/PHL-2032/2024	A	L	L	P	T	A	D	T	V	D	I
	A/Wyoming/01/2025	A	L	L	P	A	A	E	T	V	D	I
	A/Missouri/121/2024-like	T	M	Q	S	A	T	E	I	A	N	V

Supplementary Table 2.

Amino acid comparison of HA proteins among clade 2.3.2.1e HPAI A(H5N1) human isolates used in FRA

Clade	Virus	Amino acid at position:																	
		15	71	86	94	134	154	189	212	257	269	270	323	372	374	445	490	496	510
2.3.2.1e	A/Cambodia/NPH230032/2023	K	T	V	S	T	N	R	R	I	V	K	K	R	I	R	Q	R	M
	A/Cambodia/SVH240441/2024	Q	I	A	N	A	N	K	K	V	A	Q	R	K	V	K	R	K	I
	A/Cambodia/NPH230776/2023	Q	I	A	N	A	D	K	K	V	A	Q	R	K	V	K	R	R	I

Supplementary Table 3.

Amino acid comparison of HA proteins among the vaccine virus A/Astrakhan/3212/2020 and clade 2.3.2.1e HPAI A(H5N1) human isolates used in FRA

Clade	Virus	Amino acid at position:																	
		2	15	28	66	71	72	81	86	94	94	115	120	123	124	127	134	136	140
2.3.4.4b	A/Astrakhan/3212/2020	Q	Q	H	M	I	R	R	A	S	L	L	S	P	N	T	A	P	A
2.3.2.1e	A/Cambodia/NPH230032/2023	H	K	Q	L	T	N	K	V	S	F	Q	N	S	D	A	T	S	N
	A/Cambodia/SVH240441/2024	H	Q	Q	L	I	N	K	A	N	F	Q	N	S	D	A	A	S	N
	A/Cambodia/NPH230776/2023	H	Q	Q	L	I	N	K	A	N	F	Q	N	S	D	A	A	S	N

Amino acid at position:																						
141	154	155	162	163	181	183	184	185	189	192	200	212	217	218	219	223	226	236	257	266	268	269
P	N	D	I	S	S	N	A	E	N	K	V	K	S	Q	V	R	M	D	V	K	G	V
S	N	N	K	D	P	D	E	A	R	Q	I	R	P	K	I	S	I	N	I	R	E	V
S	N	N	K	D	P	D	E	A	K	Q	I	K	P	K	I	S	I	N	V	R	E	A
S	D	N	K	D	P	D	E	A	K	Q	I	K	P	K	I	S	I	N	V	R	E	A

Amino acid at position:																	
270	273	277	281	309	322	323	372	374	445	456	474	490	496	510	512	522	527
E	H	K	V	N	L	R	K	I	K	R	D	Q	R	I	T	A	A
K	N	R	I	S	Q	K	R	I	R	K	N	Q	R	M	I	V	V
Q	N	R	I	S	Q	R	K	V	K	K	N	R	K	I	I	V	V
Q	N	R	I	S	Q	R	K	V	K	K	N	R	R	I	I	V	V
